# Endoscopic Sphenoid Sinus Anatomic Considerations: A Study on 60 Cadavers

**DOI:** 10.22038/ijorl.2021.47273.2554

**Published:** 2021-07

**Authors:** Maryam Safarian, Mohammad Sadeghi, Babak Saedi

**Affiliations:** 1 *Otorhinolaryngology Research Center, Tehran University of Medical Sciences, Tehran, Iran.*

**Keywords:** Endoscopic endonasal approach, Sphenoid sinus, Pituitary gland, Skull base surgery

## Abstract

**Introduction:**

Sphenoid sinus can be considered a key element in advanced sinus and skull base surgery. Due to its importance, many researchers tried to document its characteristics and evaluate possible differences among different races and populations.

**Materials and Methods:**

This study was conducted between March 2017 and December 2018 on 60 fresh adult cadavers in Tehran Forensic Medicine Center, Tehran, Iran. The evaluated variables were distances between nasal spines, posterior wall of the sphenoid, pituitary gland, and the distance between the anterior and posterior ethmoid artery and optic nerve, which were calculated using a flexible ruler through the direct length in millimeter. Another important variable was dehiscence, which was evaluated in optic and carotid artery canals.

**Results:**

After dissecting 120 sphenoid sinuses, the carotid artery was dehiscent in 24 (20%) cases, and optic nerve dehiscence was observed in 15 (12.5%) cadavers. The mean distance between the anterior wall of the sphenoid sinus and the anterior nasal spine was determined at 73.3±1.3 mm (rang: 58.3-87 mm), and the mean distance between the anterior part of the middle of the pituitary gland and the anterior nasal spine was estimated at 81.1±1.6 mm.

**Conclusion:**

According to our finding, the dehiscence of the key structural organs may be more prevalent in the Persian sphenoid sinus, which should be considered carefully in the management of related pathologies.

## Introduction

The sphenoid sinus is an important region in sinus and skull base surgery. Proximity to the carotid artery, optic nerve, pituitary gland, and brain in adjacent areas have made working in this field a tough task. Extended usage of endoscopy in approaching skull base lesions requires the re-evaluation of the precise anatomy of the sphenoid sinus (1-10). Hemorrhagic complications, cerebrospinal fluid leakage, and blindness are possible complications, and surgery through the sphenoid sinus can lead to long-lasting disabilities (1). A better understanding of the anatomical variations of adjacent neurovascular organs may help to avoid the aforementioned complications (1). Endoscopic skull base surgery has brought us several benefits, such as clear access to surgical landmarks. These advantages have revolutionized many surgical approaches to skull base pathologies, such as pituitary tumors (2-7). Many authors have tried to define the characteristics of the sphenoid sinus (1,2, 8-15); however, some have given definitions that are not endoscopic (10), and the small sample sizes of some others have made the interpretation of the results difficult. Moreover, the variance among races requires this study to be conducted on different populations. Therefore, this study was performed to define the endoscopic anatomy of the sphenoid sinus and the adjacent structures.

## Materials and Methods

**Study subjects**

In total, 60 Iranian fresh adult cadaveric heads that had not suffered from severe trauma of the head and neck, did not have sinus or central nervous system diseases, and were older than 25 years of age were included in this study. The mean age of the cadavers was estimated at 46.7±13.3 years, and the majority of them (n=39; 65%) were male. This study was performed between March 2017 and December 2018 in Tehran Forensic Medicine Center, Tehran, Iran.

**Ethical approval**

The study protocol was approved by the Institutional Review Board of Tehran University of Medical Sciences, Tehran, Iran. Detailed information about the study was given to the family of the deceased and written informed consent was obtained from them. All parts of this study were conducted according to the Declaration of Helsinki.

**Method of dissection**

All cadavers were laid in the surgical position with heads being slightly extended and rotated 30º to the surgeon's site. Dissection began using a 0ºand 30ºrigid endoscope (Karl Störz) with a diameter of 4 mm and a length of 18 cm to find the superior turbinate and consequently the natural ostium of the sphenoid sinus. Subsequently, the ostium of the sphenoid sinus was extended to the largest up to the level of the planum sphenoidal to expose the widest surgical field under anterior endoscopic view. Furthermore, the optic nerve, intracavernous carotid artery, and adjacent structures were defined and examined. All procedures were recorded using a DVD recorder.Afterward, according to the Wigand techniques, posterior ethmoidectomy was performed to find Onodi cells, as well as anterior and posterior ethmoid arteries. All septets of the sphenoid sinus were removed. In the next step, the same procedures were performed in the opposite site and then intersphenoid septa were removed, and the pituitary gland was exposed. The internal carotid artery on both sides was dissected using a microdrill and cutting punch.

**Variables and measurements**

**Fig 1:**
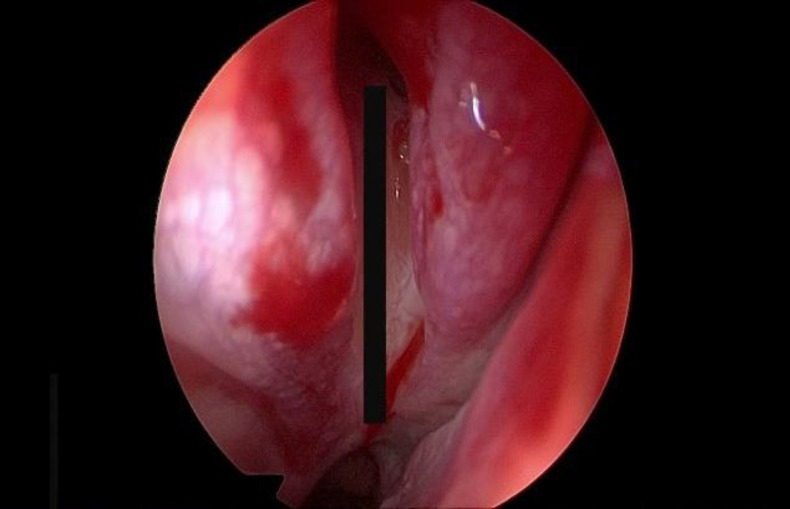
Measurement between natural ostium and the choana

In addition to demographic characteristics, the distance between nasal spines, the posterior wall of the sphenoid, pituitary gland, and the distance between the anterior and posterior ethmoid artery and optic nerve were calculated using a flexible ruler through the direct length in millimeter. Dehiscence in the carotid artery and optic nerve was recorded via direct touching of them by the tip of the suction. The rest of the variables were measured using digital videos provided by recording dissections. The angles and distances were calculated with adobe Photoshop 7 measure tools which corrected the value comparing with the plastic ruler which was placed in the field of the surgery. The shape and size of the sphenoid sinus ostium, the distance between the natural ostium, as well as the choana and the superior turbinate were calculated in this study (Figure 1). 

This study also measured the opticocarotid angle, the distance between the pituitary gland and the lower wall of the sphenoid sinus, the distance between medial margins of the cavernous carotid artery and the inferior margin of the pituitary gland, and two carotid arteries at the longest axis. 

**Statistical method**

The data were analyzed in SPSS software (version 15.0) using descriptive statistical methods (mean±SD). The sample size was calculated considering α=0.05, d=%4, and P=0.05. A p-value less than 0.05 was considered statistically significant. 

## Results

Out of 60 dissected cadavers (120 sphenoid sinuses), the carotid artery was dehiscent in 24 (20%) sphenoid sinuses. 

**Table 1:** Findings in the cadaveric study

**Findings in the cadaveric study**	**Number (%)**
Optic nerve was dehiscent	15 (12.5%)
Mean distance of optic nerve with the posterior ethmoid	8.336±2.27 mm
Mean distance of optic nerve with the anterior ethmoid	14.34±2.74 mm
Mean distance of the sphenoid sinus and the anterior nasal spine	73.3±1.3 mm
Mean distance of the anterior wall of the sphenoid sinus and the anterior nasal spine	73.3±1.3 mm

**Fig 2:**
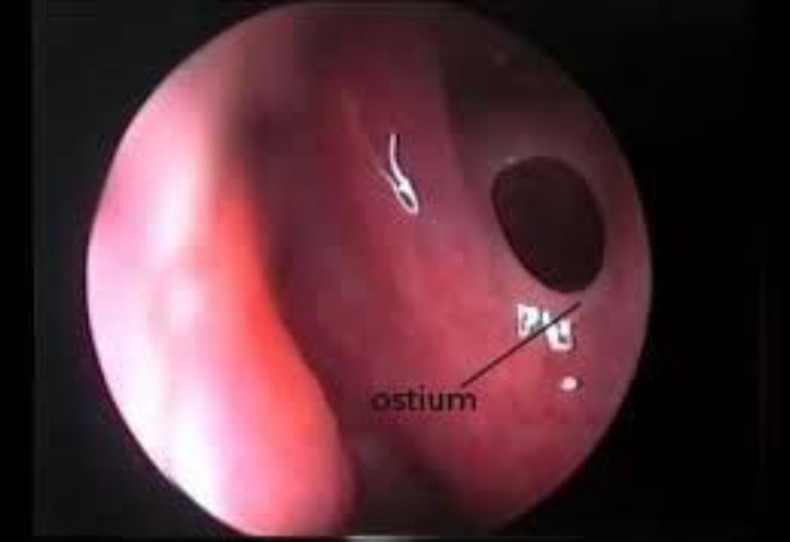
Ostium of the sphenoid sinus

The dehiscences were unilateral and bilateral in 6 (5%) and 9 (15%) sphenoid sinuses and cadavers, respectively. The optic nerve was dehiscent in 15 (12.5%) cadavers which were unilateral in 7 (5.83%) sphenoid sinuses and bilateral in 4 (6.66%) cadavers. The mean distances between the optic nerve, as well as the posterior and the anterior ethmoid artery, were 8.336±2.27 and 14.34±2.74 mm, respectively. The shapes of the sphenoid sinus ostium were circular and elliptical in 48 (40%) and 72 (60%) cadavers, respectively. Furthermore, the mean values of the longer and the shorter axis were 3.28±1.5 and 2.76±1.45 mm, respectively, with an overall mean of 3.02±1.47 mm. The shortest ostium was estimated at 1.5×1.5 mm, and the longest one was obtained at 5.5×5.5 mm (Figure. 2)

**Fig 3:**
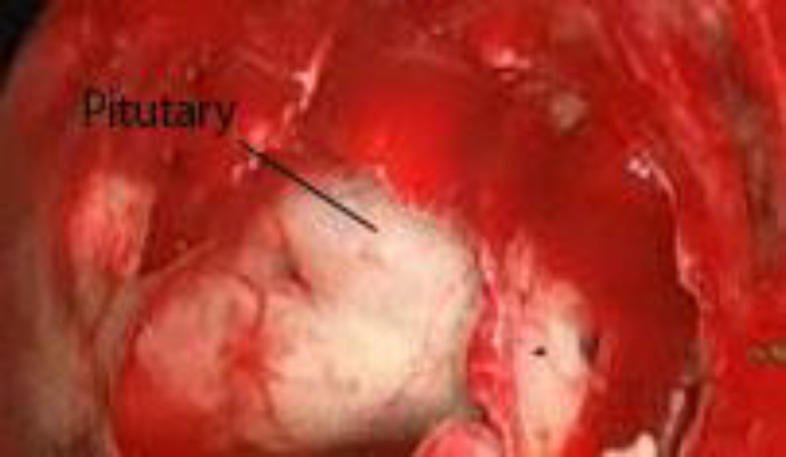
Pituitary gland

The mean distance between the anterior wall of the sphenoid sinus and the anterior nasal spine was estimated at 73.3±1.3 mm (distance range: 58.3-87 mm), and the mean distance between the anterior part of the middle of the pituitary gland and the anterior nasal spine was determined at 81.1±1.6 mm (distance range: 68-100 mm) (Figure 3).

**Fig 4:**
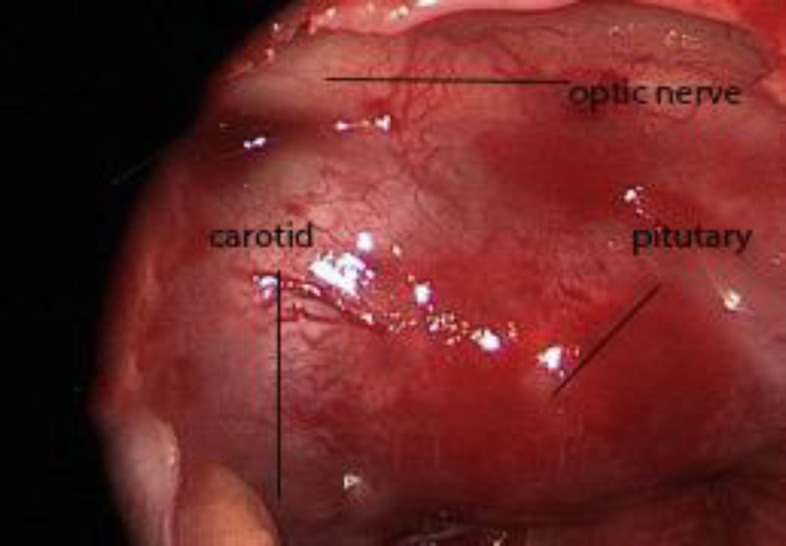
Distance between the carotid artery and the pituitary gland

The vertical distance between the inferior part of the pituitary gland and the lower wall of the sphenoid sinus was calculated at 5.89±1.38 mm (distance range: 6.5-13 mm), and the mean distance between the carotid artery and the pituitary gland was determined at 5.89±1.38 mm (distance range: 3.7-8.2 mm) (Figure 4)

The mean inter-carotid distance was estimated at 13.2±2.75 mm (distance range: 7.1-17.6 mm). Furthermore, the mean distance between the natural ostium and the posterior ch

oana was obtained at 15.34±1.83 mm (distance range: 12.3-19.8 mm). In addition, the distance between the superior turbinate and the natural ostium was calculated at 15.33±1.82 (distance range: 12.1-19.7 mm), which showed no significant relationship in this regard (t-test; P=0.98). It should be mentioned that no significant difference was observed between gender and the evaluated items. The mean opticocarotid angle was estimated at 67.96±6.04º (range: 58º-78º). In addition, the mean distances between the optic nerve, as well as the anterior and posterior ethmoid artery were determined at 14.3±2.78 and 8.38±2.78 mm, respectively. It is worth mentioning that the Onodi cells were found in 11 (18.3%) cadavers.

## Discussion

Many approaches to sinus pathologies have been modified after wide use of endoscopy in the management of sinus diseases. The expanded use of endoscopy is now far from its usual usage in paranasal sinus diseases to manage skull base lesions. Among different skull base pathologies, pituitary lesions are of great importance in which endoscopic surgery through the sphenoid sinus has made surgical approaches less traumatic (16-18).

Endoscopic skull base surgery in the vicinity of the sphenoid sinus is very difficult because of its anatomic complexity. During the past decades, many authors tried to define its surgical anatomy and make a better surgical approach to this area (1,2,4,5,8,10,11,15,19-25).

A better understanding of the anatomy of this region may improve surgical techniques and also help to avoid surgical complications during and after surgery. 

The normal distance between cavernous internal carotid arteries is a critical landmark since the detection of the boundaries of dissection in skull base surgery is a vital step to avoid complications; however, it should be considered that this distance is variable in the skull base pathologies (1). 

Despite all these difficulties, little anatomical research has been conducted concerning the anatomy of the sphenoid sinus. In our series, the rates of carotid artery dehiscence and optic nerve dehiscence were 24% and 12.4%, respectively, which were higher than those in the other studies with a reported rate of about 8% for both (16). This variation can be observed in so many reports due to the methods of assessment and the ethnic groups. 

Anusha et al. showed that in the majority of the large studies, the carotid artery could be dehiscent in about 22% of cadavers, which was consistent with our series. However, there were other reports of low rates of dehiscence in 4% of the cases; moreover, 41% and 67% of dehiscences were noted in Libyan and Asian populations in cadaveric studies, respectively (26). The Onodi cells could be found in 11 (13.8%) cadavers which was similar to those in other reports (16, 27). The mean distance between the sphenoid sinus natural ostium and the nasal spine was 73.3±1.3 mm which was relatively longer than that in some other reports, and since this distance is shorter in Asian cadavers, it is more similar to the Caucasian skulls (25). 

The distance between the superior turbinate and the natural ostium (A) was equal to the distance between the natural ostium and the superior part of the posterior choana (B) (A=B=1.5 cm). These landmarks can be used to find the ostium of the sphenoid sinus in sinus surgery. This finding was in line with the results of other reports (25).

In the presellar area, the mean inter-carotid distance was similar to that in the other reports (28). Therefore, the trans-sphenoid approach to skull base lesions can be safe and feasible; however, the awareness of the surgical anatomy and angles, as well as the ratios of this organ are mandatory. 

## Conclusion

Our findings showed that the dehiscences of the vital structures were more common in Iranian cadavers. Accordingly, this should be considered in the surgical treatment of these pathologies. 
